# BODY AWARENESS, STRESS AND SYMPTOMS IN AUTONOMIC DYSFUNCTION IN PATIENTS WITH CHRONIC PAIN: AN EXPLORATIVE STUDY

**DOI:** 10.2340/jrmcc.v7.13374

**Published:** 2024-06-24

**Authors:** Emma VARKEY, Raquel GOTTFRIDSSON, Anna GRIMBY-EKMAN, Anna BJARNEGÅRD SELLIUS, Maria ÖSTMAN, Paulin ANDRÉLL

**Affiliations:** 1 Department of Health and Rehabilitation/Physiotherapy, Institute of Neuroscience and Physiology at Sahlgrenska Academy, University of Gothenburg, Gothenburg, Sweden; 2 Region Västra Götaland, Sahlgrenska University Hospital/Östra; 3 Department of Occupational Therapy and Physiotherapy, Gothenburg, Sweden; 4 Department of Anaesthesiology and Intensive Care Medicine/Pain Centre, Gothenburg, Sweden; 5 School of Public Health and Community Medicine, Institute of Medicine, Sahlgrenska Academy, University of Gothenburg, Gothenburg, Sweden; 6 Region Västra Götaland, Närhälsan Gibraltar Rehabilitation Centre, Gothenburg, Sweden; 7 Department of Anaesthesiology and Intensive Care Medicine, Institute of Clinical Sciences at Sahlgrenska Academy, University of Gothenburg, Gothenburg, Sweden

**Keywords:** autonomic dysfunction, body awareness, chronic pain, pain intensity, pain duration, stress level

## Abstract

**Objective:**

To assess pain outcomes, stress levels and body awareness among patients with chronic pain and explore potential associations between these variables.

**Design:**

An explorative study.

**Methods:**

Patients with chronic pain in primary and specialist care were assessed regarding pain intensity using the Numerical Rating Scale (NRS; 0–10 point scale) and stress levels using the Stress and Crisis Inventory (SCI-93; 0–140). To assess body awareness, multidimensional assessment of interoceptive awareness (MAIA; 0–5), a widely used self-report measure of interoceptive bodily awareness was used.

**Results:**

Participants (*n* = 42) reported an average NRS of 4.4, elevated stress levels and low body awareness. Stress levels were moderately correlated with pain intensity (*r* = 0.53; *p* < 0.001; 95% confidence interval [CI] 0.25–0.72) and number of pain sites (*r* = 0.58; *p* < 0.001; 95% CI 0.32–0.76). The regression analysis showed that pain outcomes predicted stress level scores and explained almost 50% of variance (*R*^2^ = 0.47, *p* < 0.001). Moreover, shorter pain duration predicted a higher body awareness (*p* = 0.04).

**Conclusion:**

In patients with chronic pain, high pain intensity and multiple painful sites seem to be associated with impaired stress regulation. The patients had low body awareness, which was negatively influenced by pain duration.

LAY ABSTRACT

Many people who live with chronic pain also suffer from other coexisting health issues. Previous research has suggested that some chronic pain conditions may be linked with high stress levels and low body awareness. This study was performed in 2022 in Gothenburg, Sweden, and included 42 patients with different chronic pain diagnoses. Through questionnaires, we aimed to evaluate pain, stress and body awareness and to identify any associations between these factors. Average pain intensity was 4.4 on a 0–10 scale, and patients presented with high stress levels and low body awareness. These results suggest that pain can predict higher stress levels: the more intense and widespread the pain, the higher the stress level. The shorter the time with pain, the higher the body awareness. This knowledge can be helpful to develop and direct treatment for pain patients who also struggle with high stress levels and low body awareness.

Approximately 20% of European adults contend with moderate to severe chronic pain (1), with compelling evidence indicating comorbidities such as sleep disturbances, depression, and anxiety (2). Alterations in stress regulation have also been described through impairment in the hypothalamic–pituitary–adrenal axis, that is weakened control over the stress-response system and dysfunction in the autonomic nervous system (ANS). Clinical manifestations of this are elevated stress symptoms, such as physical and mental fatigue and sleep disturbances. The term “autonomic dysfunction” has been used to describe the phenomenon of progression from a physiological and correct stress response to pathological stress that negatively affects the patient (3–6).

Body awareness, often referred to in the literature, yet lacking a clear definition, pertains to the capacity to comprehend and interpret bodily signals (7). Body awareness is closely related to and influenced by proprioception, exteroception and interoception (8), where interoception is shown to play a role in the symptom experience of chronic pain (9). Interoception involves sensing, interpreting, and regulating internal body signals and encompasses communication between peripheral systems and the central nervous system (10). In the literature, body awareness is occasionally delineated as interoceptive awareness, wherein it is characterized as either adaptive or maladaptive (11, 12). Adaptive body awareness includes balanced attention to body signals and the capacity to move from a focus on symptoms to a state of perceptual attentive presence in one’s body. Contrary to this favorable attention to body signals, maladaptive attention is characterized by traits such as pain catastrophizing, hypervigilance and avoiding or ignoring bodily sensations (7, 13). Impaired body awareness has been described among chronic pain patients (14, 15). In addition, maladaptive body awareness has been proposed to have a negative correlation with chronic pain and increased pain severity (14, 16).

Furthermore, symptoms of central sensitization in chronic pain patients have been shown to be inversely associated with some domains of body awareness (11), and it has been suggested that there is an interaction between interoception and dysfunctional stress regulation (17–19).

From a clinical perspective, knowledge about stress levels and body awareness are important when developing a personal rehabilitation program for chronic pain patients. However, the current data on stress-related symptoms and the degree of body awareness among patients with chronic pain are uncertain, and little is known about their associations.

Therefore, this explorative study aimed to assess pain outcomes, stress levels and symptoms of autonomic dysfunction and body awareness in a population of patients with chronic pain. Additionally, the aim was to determine whether there are associations between pain, stress levels and body awareness.

## MATERIALS AND METHODS

### Study design

This explorative study is part of an investigation of the psychometric properties of a recently developed instrument aimed to measure quality of movement in chronic pain patients.

### Participants and recruitment

Patients were consecutively recruited from the Pain Centre at the Sahlgrenska University Hospital/Östra, Närhälsan Gibraltar Rehabilitation Centre and the Centre for Sexual Health in Gothenburg, Sweden over 3 months in 2022. All patients who participated in rehabilitation with one of three physiotherapists (EV, ABS, MÖ) during the study period and fulfilled the inclusion criteria were invited to participate in the study. Eligible patients had verified chronic pain and the ability to understand and speak Swedish. Exclusion criteria were patients < 18 years of age and those with considerable psychiatric comorbidity that could impact participation in the study. Patients were included regardless of the underlying chronic pain diagnosis.

### Procedure

All participants underwent an evaluation of quality of movement as part of the original study. Thereafter, they completed the following questionnaires: the Stress and Crisis Inventory (SCI-93) and the Multidimensional Assessment of Interoceptive Awareness (MAIA). Participants were also asked questions on personal characteristics and pain status, which were scored using the Numeric Rating Scale (NRS), number of pain sites and pain duration in years.

### Assessments

*Pain outcomes.* Pain locations were reported in free text and transferred to an anatomical map of 36 different pain sites, resulting in *number of pain sites*. Based on the obtained value for *number of pain sites*, a further division was made into one site only, two to six sites and seven or more sites (20). Some patients reported pain in the whole body and were entered under the ≥ 7 sites classification. The division into groups of pain sites was used in the statistical analyses and is referred to in the following sections as *pain sites*. “Pain duration” was defined as number of years suffering from chronic pain and was divided into <1 year, 1–5 years, 6–10 years and > 10 years.

The 0–10 NRS was used for assessment of self-reported pain intensity, where 0 = “no pain,” 10 = “worst pain possible” (21, 22). Participants reported pain intensity at rest and motion on the previous day and in the last week. The *NRS average* was calculated as a mean value of the reported NRS scores.

*Stress and crisis inventory.* The SCI-93 is developed to quantify self-perceived stress level and symptoms associated with autonomic dysfunction. The instrument has demonstrated good reliability, consistency and stability in detecting the severity of autonomous symptoms (23). It includes 35 items (see Table IV), which can be rated on five levels depending on the impact the item has on daily life, from 0 = “not at all,” to 4 = “very much” (23). The resulting score ranges from 0 to 140 and the following cut-offs are suggested: 0–25 normal stress reaction, 26–50 mild function impairment, 51–75 significant function impairment, 76–100 considerable function impairment, and 101–140 extensive function impairment (24).

*Multidimensional assessment of interoceptive awareness*. Body awareness was assessed using the MAIA questionnaire, version 2 (MAIA-2), which consists of 37 items divided into eight subscales, and assesses different aspects of adaptive body awareness. MAIA is the most widely used self-report measure of interoceptive bodily awareness (25). Each item can be scored from 0–5, where 0 = “never” and 5 = “always.” The *MAIA average* was calculated as a mean value of the reported MAIA scores. A higher score indicates greater body awareness. MAIA is evaluated for validity and reliability (26).

### Statistical analyses

Descriptive statistics were used; data are presented as mean, standard deviation (SD), median and range. Analyses were performed in IBM SPSS Statistics, version 25.0 (IBM Corp., Armonk, NY, USA). To characterize the overall relationships between pain outcomes, SCI-93 and MAIA correlations were calculated using Spearman’s rank correlation coefficients (*r*_s_). The interpretation of correlation coefficients was done based on the following criteria: very strong correlation (*r* = 0.90–1.00), strong correlation (*r* = 0.70–0.89), moderate correlation (*r* = 0.50–0.69), weak correlation (*r* = 0.26–0.49) and no or negligible correlation (*r* = 0.00–0.25) (27). Confidence intervals (CIs) were calculated using Fisher’s r–z transformation. To further evaluate whether scores on the SCI-93 and dimensions of the MAIA were associated with pain outcomes, multiple regression analysis models were computed, with the SCI-93 and MAIA, respectively, as dependent variables and pain outcomes (pain intensity, pain sites and pain duration) as independent variables. A *p* < 0.05 was set for statistical significance. The sample size (*n* = 42) was determined by a power analysis for the original study. Since this is an explorative study in a mixed group of chronic pain patients, which may generate hypotheses for further studies within this patient group, no further power analysis was done.

### Ethical approval

The study, as well as the supplementary application, was approved by the Swedish Ethical Review Authority (Dnr: 2021-03321 and Dnr: 2021-06169-02). All participants gave oral and written consent before they were included in the study.

## RESULTS

### Participants

**Fig. 1:**
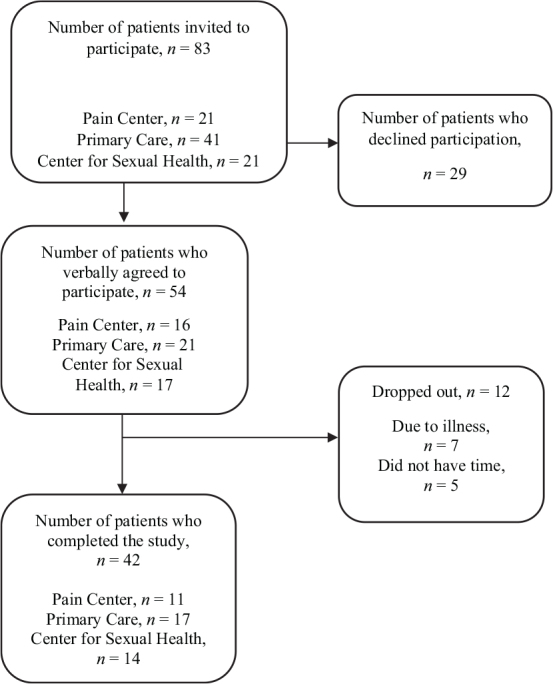
Flowchart of the study population.

A total of 54 patients initially expressed interest in participating in the study (Fig. 1). Seven patients dropped out due to flu-like symptoms in response to the restrictions in place during the COVID-19 pandemic. Data on the 42 participants who completed the study (35 women, 7 men) were collected and analysed. Distribution and demographics of the study population are shown in Table I.

**Table I:** Participant demographics and pain outcomes, by health care center

	Pain center *n* = 11	Primary care *n* = 17	Center for Sexual Health *n* = 14	All patients *n* = 42
Women/men, *n* (%)	7/4 (64/36)	14/3 (82/18)	14/0 (100/0)	35/7 (83/17)
Age, years, mean (min; max)	42 (18; 57)	45 (21; 67)	33 (27; 50)	40.5 (18; 67)
**Pain sites**, *n* (%)				
1 site only	1 (9)	3 (18)	0 (0)	4 (10)^a^
2–6 sites	2 (18)	7 (41)	10 (77)	19 (46)^a^
≥7 sites	8 (73)	7 (41)	3 (23)	18 (44)^a^
**Pain duration^a^**, *n* (%)				
<1 year	0 (0)	0 (0)	0 (0)	0 (0)
1–5 years	4 (36.4)	7 (41)	2 (15)	13 (32)
6–10 years	4 (36.4)	7 (41)	7 (54)	18 (44)
>10 years	3 (27.3)	3 (18)	4 (31)	10 (24)

a *n* = 41.

### Pain outcomes

All participants reported a pain duration of > 1 year and approximately one out of four reported pain duration > 10 years. Moreover, most participants described pain in more than one site (*n* = 37; 90%). The most prevalent pain locations were neck/shoulder (*n* = 16; 39%), back (*n* = 15; 36%) and the pelvic/genital area (*n* = 14; 34%). The average pain intensity, according to the NRS, was 4.4. A higher mean pain intensity was observed for pain in the *last week* compared with pain on the *last day,* both at rest and in motion (Table II).

**Table II:** Scores on the Numeric Rating Scale, Stress and Crisis Inventory and Multidimensional Assessment of Interoceptive Awareness (MAIA) questionnaire, by health care center

	Pain center Mean (SD)	Pain center Median (min–max)	Primary care Mean (SD)	Primary care Median (min–max)	CSH Mean (SD)	CSH Median (min–max)	Total Mean (SD)	Total Median (min–max)
**NRS score** *n = 42*								
Average score	6.5 (1.9)	7.0 (2.0–8.5)	4.7 (1.5)	4.8 (2.5–8.0)	2.4 (2.2)	2.0 (0.0–5.5)	4.4 (2.4)	4.8 (0–9)
Last day at rest	6.3 (2.0)	7.0 (2.0–8.0)	4.4 (1.5)	4.0 (3.0–8.0)	2.1 (2.4)	1.0 (0.0–6.0)	4.1 (2.5)	4.0 (0–8)
Last day in motion	6.6 (2.0)	7.0 (2.0–9.0)	4.5 (1.9)	4.0 (1.0–8.0)	2.0 (1.9)	1.5 (0.0–5.0)	4.2 (2.6)	4.0 (0–9)
Last week at rest	6.6 (2.3)	7.0 (2.0–9.0)	4.9 (1.7)	5.0 (2.0–8.0)	2.7 (2.9)	1.5 (0.0–7.0)	4.6 (2.7)	5.0 (0–9)
Last week in motion	6.6 (2.1)	7.0 (2.0–9.0)	5.0 (1.8)	4.0 (2.0–8.0)	2.9 (2.4)	3.0 (0.0–6.0)	4.7 (2.5)	5.0 (0–9)
**SCI-93 score** *n = 41*	57.5 (22.5)	52 (17–85)	50.7 (19.7)	52.5 (16–81)	48.8 (22.8)	44.0 (22–94)	52 (21)	48 (16–94)
**MAIA score** *n = 42*								
Average score	2.5 (0.7)	2.4 (1.1–3.5)	2.8 (0.7)	2.8 (1.5–4.3)	2.5 (0.7)	2.5 (1.4–3.9)	2.6 (0.7)	2.6 (1.1–4.3)
Noticing	3.5 (0.8)	3.3 (2.5–5.0)	3.3 (0.9)	3.3 (2.0–5.0)	2.8 (1.1)	2.9 (0.5–4.8)	3.2 (1.0)	3.2 (0.5–5.0)
Not distracting	1.5 (0.9)	1.8 (0.0–3.0)	2.1 (1.0)	2.0 (0.5–4.1)	1.9 (0.9)	2.0 (0.8–3.7)	1.9 (1.0)	2.0 (0.0–4.1)
Not worrying	2.1 (0.7)	2.4 (0.8–3.2)	2.3 (1.2)	1.8 (0.8–4.6)	2.2 (1.0)	2.2 (0.6–4.4)	2.2 (1.0)	2.2 (0.6–4.6)
Attention regulation	2.8 (0.9)	3.0 (1.6–3.9)	2.8 (1.0)	2.7 (1.4–4.6)	2.6 (1.0)	2.4 (0.9–4.4)	2.7 (0.9)	2.7 (0.9–4.6)
Emotional awareness	3.5 (0.9)	3.4 (1.4–5.0)	3.5 (0.8)	3.4 (1.8–4.8)	3.5 (1.1)	3.8 (1.4–5.0)	3.5 (0.9)	3.5 (1.4–5.0)
Self-regulation	2.5 (1.0)	2.5 (0.3–4.3)	2.8 (1.1)	3.0 (1.0–4.5)	2.9 (0.8)	3.2 (1.0–4.0)	2.8 (1.0)	2.8 (0.3–4.5)
Body listening	2.0 (1.2)	2.0 (0.3–3.7)	2.4 (1.3)	2.3 (0.3–4.7)	2.0 (1.3)	2.0 (0.0–4.3)	2.2 (1.3)	2.0 (0.0–4.7)
Trusting	2.1 (1.1)	2.7 (0.0–3.0)	3.1 (1.3)	3.7 (0.3–4.7)	2.4 (1.4)	2.7 (0.0–4.7)	2.6 (1.3)	2.7 (0.0–4.7)

CSH: Center for Sexual Health; NRS: Numerical Rating Scale; SCI-93: Stress and Crisis Inventory.

Higher scores on the NRS (range 0–10), SCI-93 (range 0–140) and MAIA subscales (range 0–5) indicate higher pain intensity, more stress-related autonomic symptoms and more interoceptive awareness, respectively.

### Stress and crisis inventory

Most participants reported SCI-93 scores in the range of 26–50 (*n* = 18; 43.9%) (Table III).

**Table III:** Stress and Crisis Inventory scores based on existing cut-offs for impact of stress levels on daily function All patients, *n* = 41

SCI-93 score	*n* (%)
0–25	4 (9.8)
26–50	18 (43.9)
51–75	11 (26.8)
76–100	8 (19.5)
101–140	0 (0)

SCI-93: Stress and Crisis Inventory.

A score of 0–25: normal stress reaction; 26–50: mild function impairment; 51–75: significant function impairment; 76–100: considerable function impairment; 101–140: extensive function impairment.

The total mean SCI-93 score was 52 (SD ± 21), and the median was 48 (range 16–94). Table IV describes rating outcomes of the various symptoms in the SCI-93. Table II reports SCI-93 scores by health care centre.

**Table IV:** Description of means and percent of rating outcomes (0–4, where 0 means not disturbed at all and 4 means very disturbed) of the various symptoms in the Stress and Crisis Inventory

Symptoms (patients, *n* = 41)	Mean (SD)	0 (%)	1 (%)	2 (%)	3 (%)	4 (%)
Tension in the jaws, (*n* = 41)	2.0 (1.4)	20	22	12	32	15
Muscle pain, (*n* = 40)	2.4 (1.3)	10	17	20	24	27
Muscle stiffness, (*n* = 39)	2.5 (1.1)	5	12	27	37	15
Muscle exhaustion, (*n* = 41)	2.0 (1.2)	15	15	34	24	12
General weariness, (*n* = 40)	2.7 (1.1)	2	17	17	34	27
Irritability, (*n* = 40)	1.8 (1.0)	10	32	32	22	2
Tingling sensations in the body, (*n* = 40)	1.2 (1.1)	39	22	20	17	0
Numbness in arms/hand/legs/feet, (*n* = 41)	0.9 (1.2)	59	15	10	15	2
Burning sensations in the body, (*n* = 40)	0.9 (1.2)	54	22	10	7	5
Disturbed sleep, (*n* = 41)	2.2 (1.4)	20	12	15	32	22
Irritation in the eyes, (*n* = 40)	1.3 (1.3)	39	20	20	15	5
Dry mouth, (*n* = 41)	1.1 (1.2)	44	15	12	12	2
Hypersensitivity to fragrances/lights/sounds, (*n* = 40)	1.9 (1.5)	22	24	12	17	22
Weather susceptibility, (*n* = 40)	1.5 (1.3)	29	27	12	24	5
Swelling sensation in hands/feet, (*n* = 40)	0.6 (1.1)	68	17	5	2	5
Fumbling hands/fingers, (*n* = 40)	1.0 (1.3)	46	29	7	7	7
Trembling hands, (*n* = 40)	0.5 (0.7)	63	27	5	2	0
Dizziness, (*n* = 39)	1.2 (1.1)	29	37	15	12	2
Varying loose/hard stools, (*n* = 40)	1.7 (1.3)	24	17	37	5	15
Restlessness, (*n* = 40)	2.6 (1.0)	2	10	29	41	15
Itching, (*n* = 39)	1.2 (1.2)	39	22	15	17	2
Cold hands/feet, (*n* = 39)	2.1 (1.3)	15	22	15	29	15
Cold/sweaty alternating sensations, (*n* = 40)	1.5 (1.4)	37	7	29	17	7
Frequent micturition, (*n* = 40)	1.6 (1.6)	37	20	5	17	20
Reduced concentration, (*n* = 40)	2.7 (3.5)	7	27	24	15	22
Reduced memory, (*n* = 39)	2.1 (1.4)	15	20	24	17	20
Pain in the skin when touched, (*n* = 40)	0.8 (1.2)	54	29	5	2	7
Boiling sensation in the body, (*n* = 39)	0.6 (1.1)	63	17	5	5	5
Reduced appetite, (*n* = 40)	0.7 (1.0)	59	17	12	10	0
Fever sensations without fever, (*n* = 40)	1.1 (1.1)	39	24	20	15	0
Palpitation, (*n* = 40)	1.3 (1.2)	34	22	22	15	5
Weight over the chest/heavy breathing, (*n* = 39)	1.3 (1.1)	27	32	22	12	2
Frequent headache, (*n* = 41)	2.0 (1.4)	22	12	32	12	22
Reduced libido, (*n* = 38)	2.2 (1.4)	12	20	22	15	24
Globus/sensation of lump in the throat, (*n* = 40)	0.8 (0.9)	46	32	12	7	0

0 = Not at all/ Never 1 = Little/ Rarely 2 = Moderately/ Sometimes 3 = Much/ Often 4 = Very much/Very often.

### Multidimensional assessment of interoceptive awareness

The average score for all subcategories on the MAIA for the total study population was 2.6 (SD ± 0.7) with a median of 2.6 (1.1–4.3). Mean and median scores for each subscale are presented in Table II.

### Associations between pain outcomes, stress levels and body awareness calculated through correlation analysis

A correlation analysis between the average SCI-93 and NRS scores resulted in a coefficient (*r*) of 0.53, suggesting a moderate correlation. Scores on the SCI-93 and pain sites were found to correlate moderately (*r* = 0.58). Remaining correlations (Tables V and VI) either had a weak or negligible correlation or did not reach statistical significance. When separate analyses were performed for the centres, strong correlations were found between the MAIA *attention regulation* and pain duration at the Pain Centre (*r* = -0.756) and between the MAIA *not distracting* and pain duration (*r* = -0.736) at the Centre for Sexual Health (data not shown).

**Table V:** Spearman’s rank correlation coefficients (*r*_s_) and 95% confidence intervals (CIs) between Stress and Crisis Inventory scores and pain outcomes including pain intensity scored on the Numeric Rating Scale, and Multidimensional Assessment of Interoceptive Awareness (MAIA) scores, respectively

	Correlation to SCI-93 score	
	*r*_s_	95% CI
**NRS score**		
Average score	0.53	0.25–0.72
Last day at rest	0.52	0.25–0.72
Last day in motion	0.45	0.16–0.67
Last week at rest	0.49	0.21–0.70
Last week in motion	0.45	0.16–0.67
**Pain sites**	0.58	0.32–0.76
**Pain duration**	0.40	0.09–0.64
**MAIA score**		
Average score	-0.27	-0.54–0.05
Noticing	-0.21	-0.49–0.11
Not distracting	-0.22	-0.50–0.10
Not worrying	0.12	-0.20–0.42
Attention regulation	-0.14	-0.44–0.19
Emotional awareness	-0.11	-0.41–0.21
Self-regulation	-0.14	-0.44–0.18
Body listening	-0.19	-0.48–0.13
Trusting	-0.24	-0.51–0.09

NRS: Numerical Rating Scale; SCI-93: Stress and Crisis Inventory.

Pain sites: 1 site only, 2–6 sites, ≥ 7 sites; pain duration: < 1 year, 1–5 years, 6–10 years, > 10 years.

**Table VI:** Spearman’s rank correlation coefficients (*r*_s_) between Multidimensional Assessment of Interoceptive Awareness (MAIA) scores and pain outcomes including pain intensity scored on the Numeric Rating Scale, with 95% confidence intervals (CIs)

MAIA subscale	MAIA average		Noticing		Not distracting		Not worrying		Attention regulation		Emotional awareness		Self–regulation		Body listening		Trusting	
	*r*	95% CI	*r*	95% CI	*r*	95% CI	*r*	95% CI	*r*	95% CI	*r*	95% CI	*r*	95% CI	*r*	95% CI	*r*	95% CI
**NRS score**																		
Average score	–0.14	–0.44–0.18	0.06	–0.26–0.37	–0.12	–0.42–0.20	0.15	–0.17–0.44	–0.06	–0.37–0.25	–0.19	–0.48–0.13	–0.14	–0.44–0.18	–0.17	–0.46–0.15	–0.05	–0.36–0.27
Last day at rest	–0.09	–0.39–0.23	0.09	–0.23–0.39	–0.17	–0.46–0.15	0.15	–0.17–0.45	–0.02	–0.33–0.3	–0.1	–0.4–0.22	–0.09	–0.39–0.23	–0.06	–0.37–0.26	–0.08	–0.38–0.24
Last day in motion	–0.15	–0.45–0.17	0.03	–0.28–0.34	–0.06	–0.36–0.26	0.14	–0.18–0.43	–0.08	–0.39–0.24	–0.2	–0.48–0.12	–0.15	–0.44–0.18	–0.18	–0.46–0.14	–0.07	–0.37–0.25
Last week at rest	–0.09	–0.39–0.23	0.12	–0.2–0.42	–0.19	–0.47–0.13	0.17	–0.15–0.46	–0.02	–0.33–0.29	–0.11	–0.41–0.21	–0.09	–0.4–0.23	–0.12	–0.42–0.20	–0.03	–0.34–0.29
Last week in motion	–0.2	–0.48–0.12	–0.05	–0.36–0.27	–0.08	–0.38–0.24	0.13	–0.19–0.42	–0.16	–0.45–0.16	–0.24	–0.51–0.08	–0.11	–0.41–0.21	–0.23	–0.51–0.09	–0.03	–0.34–0.28
**Pain sites**	–0.08	–0.38–0.24	–0.21	–0.49–0.12	–0.01	–0.32–0.31	0.31	–0.01–0.57	–0.12	–0.42–0.21	–0.01	–0.33–0.31	–0.004	–0.32–0.31	–0.03	–0.35–0.29	–0.17	–0.46–0.16
**Pain duration**	–0.28	–0.55–0.04	–0.31	–0.57–0.01	–0.12	–0.42–0.20	–0.01	–0.33–0.31	–0.36	–0.61– –.05	–0.12	–0.42–0.21	–0.15	–0.45–0.18	–0.21	–0.49–0.11	–0.2	–0.48–0.13

NRS: Numerical Rating Scale.

Pain sites: 1 site only, 2–6 sites, ≥ 7 sites. Pain duration: < 1 year, 1–5 years, 6–10 years, > 10 years.

### Associations between pain outcomes, stress levels and body awareness calculated through multiple linear regression

High scores on pain outcomes were associated with high SCI-93 scores and explained almost 50% of the variance in pain (*R*^2^ = 0.47; *p* < 0.001), pain sites (*p* = 0.161), pain duration (*p* = 0.040) and pain intensity (*p* = 0.068). Results from the linear regression model, using SCI-93 scores as dependent variable, are presented in Table VII.

**Table VII:** Linear regression to estimate the associations between pain outcomes, including pain intensity measured using the Numeric Rating Scale*, and Stress and Crisis Inventory scores

Pain outcome	B (SE)	*p*
**Pain sites**		
1 site only	-18.8 (9.84)	0.07
2–6 sites	-9.6 (7.67)	0.21
≥ 7 sites	0	
**Pain duration**		
1–5 years	-15.4 (7.69)	0.05
6–10 years	1.5 (6.57)	0.82
> 10 years Pain intensity, NRS*	0 2.8 (1.49)	0.07

NRS: Numerical Rating Scale.

* Range 0–10, where 0 = “no pain,” and 10 = “the worst pain possible.”

Pain outcomes were inversely associated with MAIA scores, explaining 14% of the variance in pain outcomes (*R*^2^ = 0.142, *p* = 0.349), pain sites (*p* = 0.821), pain intensity (*p* = 0.571) and pain duration (*p* = 0.072). Results from the linear regression model, using MAIA scores as dependent variable, are presented in Table VIII.

**Table VIII:** Linear regression to estimate the associations between pain outcome, including pain intensity measured using the Numeric Rating Scale*, and Multidimensional Assessment of Interoceptive Awareness (MAIA) total score

Pain outcome	B (SE)	*p*
**Pain sites**		
1 site only	–0.173 (0.446)	0.68
2–6 sites	–0.192 (0.32)	0.56
≥7 sites	0	
**Pain duration**		
1–5 years	0.673 (0.32)	0.04
6–10 years	0.597 (0.28)	0.04
>10 years Pain intensity, NRS*	0 –0.036 (0.06)	0.57

NRS: Numerical Rating Scale.

* Range 0–10, where 0 = “no pain,” and 10 = “the worst pain possible.”

## DISCUSSION

The results from the current explorative study suggest that levels of perceived stress and body awareness are affected by chronic pain in a group of patients with various pain conditions. The study also highlights associations between symptoms of autonomic dysfunction and both pain intensity and number of pain sites. Associations were also found between pain duration and body awareness.

The study results are important from a clinical point of view, as they highlight the importance of evaluating autonomic dysfunction and awareness in patients with chronic pain conditions and including treatments to target these areas. The results on the SCI-93 imply higher perceived stress levels in our patients (mean SCI-93 score=52; SD ±21) compared with mean scores in a healthy Swedish population (28±10). These values are considered to correlate to a significant impact on function, with limitations in occupational functioning (24). Results on the SCI-93 also support our hypothesis and are in line with previous research on elevated stress levels among sufferers of chronic pain (23, 28).

Regarding the MAIA questionnaire, the reported scores showed lower values compared with a healthy North American sample (29), indicating a generally lower body awareness among this chronic pain population. Our results align with results reported by Ciaramella et al. (30), who compared pain-free individuals with patients with recurrent and chronic pain. Interoception and body awareness appear to be relevant in pain rehabilitation, and high interoceptive awareness may be associated with a higher incidence of pain relief and improvement after therapy (31). Additionally, interventions aimed to increase interoception are reported to have positive effects on chronic pain and associated conditions, such as depression and anxiety (32).

In a population of patients with various chronic pain conditions, our analysis highlighted two important associations: firstly, elevated stress levels are associated with pain intensity and the number of pain sites. Secondly, lower body awareness is associated with pain duration. The first association regarding elevated stress response, pain intensity and number of pain sites is in agreement with the results reported by Nordeman et al., who compared patients with both chronic low back pain (CLBP) and chronic widespread pain (CWP) with patients who only suffered from CLBP. Their results suggested that higher scores would be seen on the SCI-93 when pain is more widespread (28), which is also confirmed by Ericsson et al. (23). In our results, it is not only the number of pain sites but also pain intensity and pain duration that appeared to influence autonomic dysfunction. Continuous exposure to intense, widespread nociceptive pain over prolonged periods could presumably elevate the risk that pain will develop into nociplastic pain (33, 34), often accompanied by sleep and mood disorders, as well as decreased quality of life (35). These changes in the pain nervous system may also be connected to associated autonomous symptoms, thus increasing SCI-93 scores (36). Dysfunction of the autonomous system has been described in widespread pain syndromes, particularly nociplastic pain, such as fibromyalgia (37), and has also been suggested to underlie CLBP (38) and endometriosis (39). Our study also suggests that autonomic dysfunction may play a role in a large group of patients with chronic pain.

While we did not find considerable correlations between pain outcomes and body awareness through the MAIA questionnaire, we found that lower body awareness in our participants was associated with pain duration. In patients seeking specialist care (Pain Centre and Centre for Sexual Health), the correlations between scores on the MAIA subscales *attention regulation* and *not distracting*, and pain duration were strong. Furthermore, the regression analysis suggests that a longer pain duration increased the probability of scoring low on the MAIA. There may have been differences between the patients recruited from primary care and from specialist care in our study, and this may have affected the relationships between body awareness and pain. We hypothesize that psychiatric comorbidity was more common in patients seeking specialist care (40) and that these patients were more likely to engage in catastrophizing. However, these aspects were not assessed in this study. Lower levels of body awareness and higher levels of pain catastrophizing have been shown to have a negative effect on pain habituation (41). In addition, adaptive body awareness may mediate the relationship between the symptoms of central sensitization and pain intensity by reducing reactivity in the limbic system (11). It is possible that because of the physiological changes seen in chronic pain patients, maintaining adaptive body awareness is more difficult as pain duration increases (42). Body awareness may therefore both affect the development of chronic pain and be affected by chronic pain.

Although we saw some associations between the measured variables in our study, the relationship between these variables is not clear, which highlights the need for further research. From a clinical perspective, both multidisciplinary evaluations and treatment in patients with severe chronic pain would appear to be important. We suggest, based on our findings, that the evaluation of stress levels and body awareness should be included when evaluating the effects of chronic pain, especially in patients with several pain locations, high pain intensity and long pain duration. This may play an important role in developing a person-centered rehabilitation plan and may be valuable in selecting the components of rehabilitation that most benefit the individual patient.

### Strengths and limitations

The broad inclusion criteria of all chronic pain conditions, regardless of underlying diagnosis, can be viewed as a study strength. Consequently, we believe that the heterogeneity of the sample allows generalization to a wider population. However, this could also be considered a limitation, as different disease populations would presumably differ in the questionnaires used. Furthermore, the final sample size of 42 participants is relatively small and caution should be used when interpreting the results. With a larger study sample, between-group analysis would be more useful in showing the potential impact of the underlying diagnosis and correcting for other possible influencing factors. The small sample size may also have contributed to the limited correlations between MAIA and pain outcomes, while other studies with larger study populations demonstrated better correlations (11). The sample size was, however, restricted by the study on quality of movement. Another potential influencing factor is that the study population included patients from the Centre for Sexual Health. These patients generally had lower mean scores in pain intensity, which may have had an impact on the correlations. The method used to evaluate pain (questions about intensity over last day and week) does not capture all aspects of living with pain. In patients from the Centre for Sexual Health, the burden of pain may present differently (e.g. neck pain or back pain), causing avoidance in important domains in life, rather than causing a continuous high pain intensity. These may explain cases where lower values are seen on the NRS, while high values are seen for stress symptoms.

### Conclusion

The findings in this study suggest that patients with high pain intensity and multiple pain sites experience increased stress levels. Chronic pain patients present with elevated stress levels and substantial autonomic symptoms (as reported on the SCI-93), indicating a significant impact on function in daily life.

Moreover, it appears that patients with chronic pain exhibit low body awareness and a negative association was found between body awareness and pain duration in this study. Further research is needed to investigate the associations between pain, stress level and body awareness and the effects of treatments aiming to decrease stress levels and improve body awareness in chronic pain patients.

## Trial details

The original study was registered in the Swedish research web FoU i VGR (project number 274353).
